# An Overview of Genetics of Moyamoya: Beyond *RNF213* Gene

**DOI:** 10.3390/ijms27104431

**Published:** 2026-05-15

**Authors:** Giovanni Sorte, Mariagiovanna Cantone, Rita Bella, Michele Salemi, Marialuisa Zedde, Mario Zappia

**Affiliations:** 1 Department of Medical and Surgical Sciences and Advanced Technologies “G. F. Ingrassia”, University of Catania, 95124 Catania, Italy; sorteg98@gmail.com (G.S.); rbella@unict.it (R.B.); m.zappia@unict.it (M.Z.); 2 Unit of Neurology, Policlinico University Hospital “G. Rodolico-San Marco”, 95123 Catania, Italy; 3 Oasi Research Institute-IRCCS, 94018 Troina, Italy; msalemi@oasi.en.it; 4 Neurology Unit, Stroke Unit, Azienda Unità Sanitaria Locale-IRCCS di Reggio Emilia, 42123 Reggio Emilia, Italy; marialuisa.zedde@ausl.re.it

**Keywords:** Moyamoya syndrome, RNF213, vascular genetics, ACTA2, rare diseases, molecular pathogenesis

## Abstract

Moyamoya angiopathy (MMA) is a rare, chronic progressive cerebrovascular condition characterized by bilateral stenosis or occlusion of the terminal internal carotid arteries and their major branches. This progressive occlusion triggers the development of telangiectatic and fragile vessels at the base of the brain, creating the characteristic angiographic appearance of a “puff of smoke.” Depending on the etiology, MMA is classified as Moyamoya Disease (MMD) when idiopathic and primary or Moyamoya Syndrome (MMS) when associated with underlying systemic conditions. While the *RNF213* gene, particularly the p.R4810K variant, is recognized as the major susceptibility locus for MMD in East Asian populations, it does not fully account for the global genetic landscape or the phenotypic diversity of the disease. This review provides a comprehensive overview of the genetic architecture of the entire MMA spectrum, exploring loci beyond *RNF213*. We analyze the role of genes involved in vascular smooth muscle cell contractility (*ACTA2*, *MYH11*), TGF-β signaling, and DNA repair mechanisms that drive MMS, alongside the genetic basis of syndromic forms associated with neurofibromatosis type 1, trisomy 21, and RASopathies. Understanding these diverse genetic drivers is crucial for early diagnosis, risk stratification, and the development of targeted molecular therapies.

## 1. Introduction

Moyamoya angiopathy (MMA), characterized by the spontaneous, progressive occlusion or stenosis of the distal internal carotid arteries and their proximal branches, was first described in Japan in the 1960s as Moyamoya disease (MMD). Since then, its definition has evolved from a regional vascular anomaly to a complex, globally recognized clinical entity [1]. The disease is significantly more prevalent in East Asian populations; in Japan, the estimated prevalence is approximately 3 to 10.5 per 100,000 individuals, with a noted female preponderance [2]. By contrast, the condition is approximately ten times less common in Europe and North America, with an overall U.S. incidence estimated at roughly 0.086 to 0.09 per 100,000 [3]. Hemiplegia, seizures, sensory impairment, and headaches are the primary presentations in childhood, largely resulting from cerebral ischemia. Conversely, in patients older than 30 years, the rupture of fragile collateral vessels frequently leads to intracranial hemorrhage [4]. While most cases appear to be sporadic, approximately 10% occur as familial cases, primarily within Asian populations [5].

**Figure 1:**
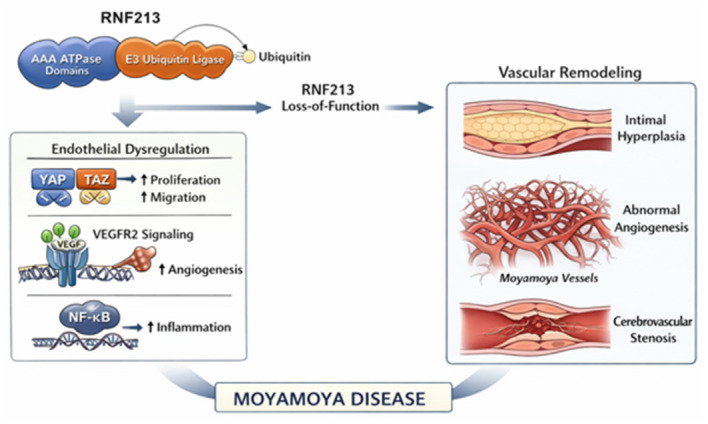
Schematic representation of RNF213 protein domains and proposed pathogenic mechanisms in Moyamoya angiogenesis. The diagram highlights the key functional units, including the AAA + ATPase core and the E3 ubiquitin ligase domain, where the founder variant p.R4810K is located and the involvement of *RNF213* in several pathways including endothelial dysregulation, aberrant angiogenesis through the VEGFR2 pathway and its interaction with inflammatory signaling pathways.

In 2011, the ring finger protein 213 gene (*RNF213*) was identified as the major susceptibility gene for MMD, particularly within East Asian populations. Located on chromosome 17 (17q25.3), it encodes the protein mysterin, a large multidomain protein with both E3 ubiquitin ligase and AAA+ ATPase activities, which plays a critical role in vascular development, angiogenesis, and the cellular response to inflammatory and hemodynamic stress (Figure 1).

Loss-of-function alterations in *RNF213*, most notably the founder polymorphism p.Arg4810Lys (R4810K), appear to deeply disrupt cerebrovascular homeostasis through mechanisms that diverge from conventional atherosclerosis [6]. Recent evidence indicates that *RNF213* mutations drive a distinct vascular phenotype characterized by a low atherosclerotic burden; indeed, carriers of the p.R4810K variant who present with intracranial arterial stenosis (IAS) exhibit significantly fewer classical atheromatous plaque features compared to their non-carrier counterparts [7,8,9]. High-resolution magnetic resonance imaging studies have further elucidated this morphological distinction. These investigations demonstrate that *RNF213*-associated steno-occlusive lesions typically lack the eccentric plaque distribution and the significant contrast enhancement characteristic of atherosclerotic inflammation, pointing instead toward a primary genetic dysregulation of intimal hyperplasia, concentric vascular remodeling, and local hemodynamics [10].

Recent evidence suggests that the genetic susceptibility associated with *RNF213* extends beyond occlusive phenomena, affecting the overall structural integrity of the vessel wall. As synthesized in a recent systematic analysis [11], there is a significant correlation between *RNF213* polymorphisms and spontaneous intracranial artery dissection.

However, this variant exhibits remarkably low penetrance, with only about 0.5% of heterozygous carriers developing the disease [12]. Conversely, the risk of disease manifestation increases to 78% in homozygous individuals, who typically present with an earlier onset and a more severe clinical phenotype [13,14]. Despite its central pathogenic role, the incomplete penetrance of *RNF213* variants, the anatomically restricted intracranial phenotype, and the clinical occurrence of MMA in *RNF213*-negative cohorts collectively underscore the profound genetic and etiological heterogeneity of the condition. This indicates that additional genetic and environmental modifiers are likely to shape disease onset and progression. Consequently, *RNF213* represents a key node within a broader, multifactorial pathogenic network rather than the sole deterministic driver of MMA. These observations suggest that in most heterozygous individuals, at least one additional genetic and/or environmental factor is required for MMD to manifest, firmly supporting a “two-hit hypothesis.” According to this model, while the *RNF213* variant represents the first “hit” (genetic predisposition), a second “hit”, such as inflammation, viral infection, or environmental stressors, is necessary to trigger the progressive steno-occlusive process characteristic of the disease [15,16,17].

Beyond *RNF213*, the identification of several non-syndromic susceptibility genes has unmasked convergent molecular cascades. These encompass the dysregulation of vascular smooth muscle cell (VSMC) proliferation and migration, aberrant endothelial signaling, pathological extracellular matrix (ECM) reorganization, and impaired nitric oxide (NO) homeostasis. Collectively, these findings suggest that MMA arises not from the isolated disruption of a single molecular axis, but from the synergistic perturbation of interconnected vascular regulatory networks, likely triggered by the aforementioned secondary genetic or environmental “hits.”

Traditionally, a critical distinction is made between MMD, considered an isolated, idiopathic condition frequently associated with the *RNF213* susceptibility gene, and Moyamoya Syndrome (MMS), where the characteristic angiographic pattern occurs in association with well-recognized underlying systemic conditions, infections, or radiation exposure. Historically, one of the main differences between MMD and MMS was considered to be the bilateral or unilateral involvement of the internal carotid artery, with MMD generally presenting as a bilateral disease and MMS as a unilateral disease. However, this assumption is increasingly difficult to support as MMS cases with bilateral involvement and MMD cases with unilateral involvement at baseline that progress to bilateral involvement during follow-up are frequently reported. The “two-hit hypothesis” has been integrated into the embryological background of MMA, as recently detailed from a neurovascular point of view, encompassing this distinction between MMS and MMD [18]. In addition, it includes the possibility of an associated systemic arteriopathy [19].

Despite the prominence of *RNF213*, a significant proportion of MMA cases—particularly in Western populations—remains genetically unexplained, necessitating a systematic exploration of alternative molecular drivers. This review aims to dissect these emerging genetic factors, providing a molecular roadmap of the non-*RNF213* landscape in MMS. For the purpose of clarity and conceptual consistency in this review, we refer to MMD only in association with *RNF213* polymorphisms, and we utilize MMA for all other conditions described in detail, encompassing the most recognized MMS cases (i.e., MMA manifesting within the context of other underlying systemic diseases).

## 2. Methods

This article was designed as a narrative review to synthesize current knowledge on the genetic landscape of MMA. A comprehensive literature search was conducted across the PubMed, Scopus, and Web of Science databases to identify relevant studies published up to January 2025. The search strategy employed a combination of keywords and Boolean operators, including the following: “Moyamoya disease”, “Moyamoya syndrome”, “Moyamoya angiopathy”, “genes”, “rare variants”, and “molecular mechanisms”. Studies were eligible for inclusion if they provided original data on genetic associations with MMA, identified novel pathogenic variants, or described clinical genotype–phenotype correlations. Additionally, the reference lists of all identified articles were manually cross-referenced to ensure the inclusion of further relevant studies. Exclusion criteria included case reports with insufficient genetic documentation and studies not available in English. Although the primary focus of this review is human pathology, select animal model studies were included only to elucidate the underlying pathophysiological pathways of specific genes. While a PRISMA-compliant systematic approach would have allowed for a rigid quantitative analysis, a narrative framework was selected as the most effective method to broadly integrate and critically discuss the rapidly evolving clinical and genetic perspectives on Moyamoya.

## 3. Results: Genes Beyond RNF213

The genetics of MMA beyond *RNF213* is characterized by significant molecular heterogeneity, with multiple genetic drivers converging on shared vascular pathways. Table 1 highlights the implicated genes according to their pathogenic mechanisms, such as, vascular smooth muscle cell (VSMC) contractile dysfunction, and nitric oxide (NO) signaling pathways. Furthermore, this table offers an integrated perspective on the relationship between genetic alterations and clinical phenotypes.

### 3.1. Genes Involving VSMC Contractile Apparatus Dysfunction

Pathogenic variants in genes regulating vascular smooth muscle cell (VSMC) contractility and arterial wall integrity represent a critical subgroup of non-*RNF213* genetic drivers of MMA. The integrity and contractile function of VSMCs are paramount for maintaining cerebrovascular homeostasis, as these cells must dynamically respond to hemodynamic stress. Mutations in genes such as *ACTA2* (Actin Alpha 2, Smooth Muscle), *MYH11* (Myosin Heavy Chain 11), and *DIAPH1* (Diaphanous Related Formin 1) directly impair the cytoskeletal architecture and the cell’s mechanotransductive capabilities, triggering a maladaptive remodeling process.

*ACTA2* encodes the smooth muscle cell (SMC)-specific isoform of α-actin, a major component of the contractile apparatus. A large family-based analysis showed that heterozygous missense mutations in *ACTA2*, initially identified in families with thoracic aortic aneurysms and dissections, are also associated with premature coronary artery disease and ischemic stroke, including cases fulfilling diagnostic criteria for MMA [20]. Specifically, variations affecting the Arg258 residue (e.g., p.R258C/H), are strongly associated with a distinctive, early-onset steno-occlusive cerebral arteriopathy [20,21,22,23]. Unlike classic idiopathic MMD, *ACTA2*-related MMA often presents as part of a systemic “smooth muscle dysfunction syndrome,” encompassing coronary artery disease and thoracic aortic aneurysms [20,24].

At the molecular level, these mutations exert a dominant-negative effect on actin filament assembly, which in turn alters the MRTF–SRF-dependent transcriptional programs. A key feature of this dysfunction is the dysregulation of TGF-β signaling. In mutation carriers, increased TGF-β bioavailability promotes a “phenotypic switch” where VSMCs transition from a quiescent, contractile state to a hyperproliferative, synthetic phenotype [24]. Pathological examinations confirm that luminal stenosis in these patients is driven by marked fibrocellular intimal proliferation and medial SMC accumulation, rather than atherosclerotic lipid deposition. This TGF-β-mediated proliferation represents the mechanistic bridge between the molecular actin defect and the macroscopic “Moyamoya-like” occlusive phenotype.

Similarly to *ACTA2*, *MYH11* is essential for the mechanical stability of the vessel wall. While MYH11 mutations primarily manifest as a systemic dilative-dissecting phenotype (e.g., Thoracic Aortic Aneurysm and Dissection), rare cases have linked it to intracranial occlusive disease [25]. In a pediatric case, a heterozygous *MYH11* missense variant was associated with progressive terminal ICA stenosis and the development of basal collateral vessels consistent with MMS [26]. Conversely, Larson et al. (2020) reported bilateral intracranial arterial stenosis in a young adult harboring an MYH11 mutation without the formation of typical Moyamoya collaterals during follow-up [27]. These observations suggest that defects in the heavy chain of the contractile unit may induce a structural fragility that, depending on the specific mutation and hemodynamic environment, can manifest as either vessel rupture or compensatory stenotic remodeling.

Recently, *DIAPH1* has emerged as a crucial candidate gene for MMS, particularly in non-Asian populations [28]. *DIAPH1* encodes mDia1, a formin family member that acts as a primary effector for Rho GTPases, playing a fundamental role in both actin polymerization and microtubule stabilization. The mechanistic insight into *DIAPH1*-related MMA lies in the disruption of the Rho/ROCK signaling axis. Pathogenic variants lead to an impaired regulation of the actin cytoskeleton, which compromises focal adhesion turnover and VSMC migration [29]. This cytoskeletal instability mirrors the defects seen in *ACTA2* mutations, further supporting a model where impaired mechanosensing at the cellular level triggers the intimal hyperplasia characteristic of MMA. By disrupting the stability of the actin–microtubule interface, *DIAPH1* variants prevent VSMCs from maintaining the vascular tone required to withstand the high-pressure environment of the carotid terminus.

### 3.2. Genes Involving Nitric Oxide Signaling and cGMP Pathway

A second critical mechanistic cluster involves the nitric oxide (NO) signaling pathway. The Guanylate Cyclase 1 Soluble Subunit Alpha 3 (*GUCY1A3*) gene encodes the α1 subunit of soluble guanylyl cyclase (sGC), a key enzyme that mediates vasodilation. sGC is the principal intracellular receptor for nitric oxide (NO) and catalyzes the conversion of GTP to cyclic GMP (cGMP), a second messenger that mediates vascular smooth muscle relaxation, inhibits SMC proliferation, and contributes to platelet and endothelial homeostasis. Loss-of-function variants in *GUCY1A3* reduce cGMP levels, leading to impaired vascular relaxation and promoting an environment conducive to VSMC hyperplasia. Functional analyses demonstrated markedly reduced or abolished sGC activity in mutant cells, leading to impaired NO-induced cGMP production. From a pathophysiological perspective, defective cGMP signaling is expected to promote impaired vasodilation and altered SMC behavior, favoring progressive arterial narrowing. Biallelic mutations in *GUCY1A3* have been identified in families presenting with MMS associated with esophageal achalasia. In the cohort described by Hervé et al. [30], affected individuals exhibited progressive stenosis of the terminal ICAs and their proximal branches, with the development of typical Moyamoya collateral networks and recurrent early-onset ischemic strokes. Importantly, the same NO-dependent pathway mediates inhibitory neurotransmission within the enteric nervous system; its disruption provides a mechanistic explanation for the associated achalasia due to failure of lower esophageal sphincter relaxation. Thus, this study supports a unifying model in which primary impairment of NO-dependent smooth muscle signaling underlies both cerebral arteriopathy and esophageal dysmotility. Collectively, these observations highlight defective vasodilatory signaling, rather than solely structural contractile abnormalities or endothelial dysregulation, as a potential driver of Moyamoya pathogenesis, expanding the mechanistic spectrum of genes implicated in the disease [30].

### 3.3. SAMHD1-Associated Autosomal Recessive Cerebral Vasculopathy

Bi-allelic loss-of-function variants in *SAMHD1* are associated with an autosomal recessive cerebral vasculopathy, typically manifesting as early-onset stroke, multifocal stenosis of major intracranial arteries, and an MMA pattern in numerous affected individuals. In a founder Old Order Amish cohort, patients exhibited recurrent ischemic strokes starting in childhood or early adolescence, frequently resulting in permanent neurological deficits [31]. Pathophysiologically, these mutations lead to a near-total loss of *SAMHD1* protein expression and are linked to a constitutive type I interferon (IFN) signature, a hallmark of Aicardi–Goutières syndrome [32]. This suggests that IFN-mediated chronic inflammation and disrupted vascular homeostasis drive this specific form of MMA [31]. Angiographic evaluations revealed progressive steno-occlusive disease of the ICAs with concomitant collateral vessel formation, alongside chronic ischemic changes, aneurysms, and sporadic hemorrhagic events [31].

### 3.4. MTHFR Variants and Hyperhomocysteinemia

Variants in the *MTHFR* gene have been proposed as potential susceptibility factors for MMS through their critical role in homocysteine metabolism. A genome-wide association study, involving large case–control cohorts, identified the SNP rs9651118 within MTHFR significantly associated with MMS risk [33]. Notably, this variant correlates with markedly elevated serum homocysteine levels among affected individuals, a biochemical profile consistent with the diagnostic criteria for type III homocystinuria. This condition is potentially associated with thrombotic manifestations and syndromic Moyamoya patterns, suggesting a functional link between impaired one-carbon metabolism and cerebrovascular pathology. Since *MTHFR* encodes 5,10-methylenetetrahydrofolate reductase, a key enzyme in folate metabolism responsible for the remethylation of homocysteine to methionine, reduced enzymatic activity leads to hyperhomocysteinemia. This metabolic derangement is known to promote endothelial dysfunction, oxidative stress, and abnormal vascular remodeling. Such mechanisms align with the pathological features of MMA, including progressive arterial stenosis and compensatory angiogenesis. Although *MTHFR* variants are unlikely to represent primary causative mutations, their association with altered homocysteine metabolism supports the hypothesis that metabolic vascular stress may contribute to disease susceptibility or modify the clinical phenotype [33].

## 4. Syndromic Moyamoya

Cumulative evidence suggests that MMA can manifest as a secondary phenomenon within broader syndromic presentations, primarily driven by a diverse array of monogenic factors. Recent advancements in next-generation sequencing (NGS) have expanded the repertoire of Mendelian disorders where MMA features are recognized as core phenotypic manifestations. However, a critical distinction must be maintained: while these syndromic contexts often exhibit cerebrovascular involvement, many do not strictly adhere to the standardized angiographic diagnostic criteria required for a definitive diagnosis of MMA. This phenotypic mimicry underscores the complexity of classifying secondary MMAs, where the underlying genetic landscape may drive vascular remodeling that resembles, but does not fully replicate, the classic Moyamoya pattern. Table 2 summarized the main syndrome associated with MMA.

### 4.1. Down Syndrome

Individuals with Trisomy 21 (Down syndrome) exhibit a significantly increased risk of developing MMA, with an incidence estimated to be 26 to 100 times higher than that of the general population [34]. Pathogenesis is thought to be driven by a gene-dosage effect resulting from the triplication of specific loci on chromosome 21. Among these, the overexpression of *DYRK1A* (Dual-specificity tyrosine-phosphorylation-regulated kinase 1A) plays a pivotal role in predisposing patients to pathological vascular remodeling [35,36,37]. *DYRK1A* is a key regulator of angiogenesis and cell proliferation; its up-regulation has been shown to disrupt vascular smooth muscle cell (VSMC) homeostasis and promote the pro-fibrotic signaling pathways that characterize steno-occlusive lesions. Furthermore, the presence of the interferon receptor gene cluster (*IFNAR1*, *IFNAR2*, *IFNGR2*, and *IL10RB*) on chromosome 21 suggests that Trisomy 21 may also represent a localized state of constitutive type I interferon signaling. This “interferonopathy” could provide a shared mechanistic link with other genetic forms of MMA, such as *SAMHD1* mutations, where chronic inflammatory stress exacerbates endothelial dysfunction and intimal thickening [38].

### 4.2. Sickle Cell Disease

A distinct and more complex clinical scenario is presented by Sickle Cell Disease (SCD), caused by mutations in the *HBB* gene. While SCD patients frequently develop an MMA pattern, this intracranial involvement represents a secondary, often acquired, manifestation rather than a primary genetic arteriopathy. The development of MMA in SCD involves chronic hemolysis and sustained endothelial activation mediated by various genetic modifiers [39]. However, from a diagnostic and morphological perspective, the intracranial vasculopathy in SCD typically exhibits only a superficial resemblance to idiopathic MMD. Instead of the intrinsic vessel wall pathology seen in MMD, the vascular network in SCD reflects a chronic compensatory response to acquired, progressive steno-occlusive disease [40].

### 4.3. Marfan Syndrome

Marfan syndrome, a highly penetrant autosomal dominant connective tissue disorder, is caused by mutations in the *FBN1* gene (15q21.1), which encodes the extracellular matrix glycoprotein fibrillin-1 [41]. The structural defect in fibrillin-1 not only weakens the extracellular matrix (ECM) but also leads to the dysregulation and excessive bioavailability of TGF-β. While the cardiovascular hallmarks of Marfan syndrome are typically aortic root dilation and dissection, aberrant TGF-β signaling profoundly alters vascular remodeling dynamics throughout the entire arterial tree. The occurrence of MMA in Marfan syndrome is an infrequent event, reported in isolated clinical case rather than established through epidemiological data [42]. In these instances, elevated TGF-β levels are thought to stimulate maladaptive ECM deposition, intimal fibrosis, and VSMC migration, paradoxically resulting in severe steno-occlusive cerebrovascular disease rather than aneurysmal dilation [42].

### 4.4. Pseudoxanthoma Elasticum

Pseudoxanthoma elasticum (PXE) is an autosomal recessive metabolic disorder characterized by the progressive ectopic mineralization and fragmentation of elastic fibers. This condition is primarily driven by biallelic mutations in the *ABCC6* gene, which encodes a transmembrane ATP-binding cassette transporter crucial for the cellular efflux of ATP [43]. The resulting severe deficiency in the inhibitor inorganic pyrophosphate (PPi) facilitates the pathological calcification of the internal elastic lamina in medium and large arteries. The intracranial vascular involvement associated with ABCC6 mutations is notably heterogeneous, encompassing a broad spectrum ranging from small vessel disease and diffusing intracranial arterial narrowing to the formation of intracranial aneurysms and premature stroke [44,45]. In pediatric and young adult patients, the disruption and calcification of the elastic lamina may trigger a robust, compensatory fibrocellular intimal proliferation in the distal ICAs. In some instances, this process culminates in an early-onset and particularly aggressive form of MMA [46].

### 4.5. Neurofibromatosis Type 1 and Other RASopathies

Neurofibromatosis type 1 (NF1) is a common autosomal dominant disorder frequently associated with MMS. The condition arises from mutations in the *NF1* gene (17q11.2), which encodes neurofibromin, a critical negative regulator of the RAS/MAPK signaling pathway [47]. A loss of functional neurofibromin leads to hyperactive RAS signaling, promoting VSMC hyperproliferation and subsequent intimal hyperplasia. While often classified under the umbrella of MMS, it is important to note that the vascular involvement in NF1 is notably heterogeneous and frequently does not strictly satisfy the full angiographic diagnostic criteria for MMA. Furthermore, MMA is not the sole intracranial vascular manifestation of the disease; NF1-associated vasculopathy encompasses a broader spectrum of anomalies, including intracranial aneurysms and focal stenoses in atypical locations [48,49,50]. Although the prevalence of a Moyamoya-like pattern in NF1 patients is estimated between 0.6% and 2%, it remains a leading cause of pediatric ischemic stroke in this population, necessitating vigilant neurovascular screening and a personalized diagnostic approach that accounts for its distinct clinical behavior [49,50].

Beyond *NF1*, other RASopathies—including those involving variants in *PTPN11*, *SOS1*, and *BRAF*—have been increasingly linked to the development of MMA [51]. These conditions share a common pathophysiological theme: the dysregulation of the RAS/MAPK signaling pathway, which is a critical regulator of VSMCs proliferation and endothelial function. In these contexts, the hyperactivation of this pathway drives the progressive stenotic remodeling of the ICAs, leading to the characteristic angiographic appearance of MMA. *CBL* (Cbl Proto-Oncogene) has recently emerged as a significant genetic driver of early-onset MMA. *CBL* mutations are associated with a Noonan-like syndrome and predispose individuals to juvenile myelomonocytic leukemia. Interestingly, pediatric patients harboring de novo *CBL* variants often present with aggressive, progressive intracranial arteriopathy even in the absence of full syndromic features [52,53].

### 4.6. Tuberous Sclerosis

Tuberous Sclerosis (TSC) is a multisystem neurocutaneous disorder caused by loss-of-function mutations in either the *TSC1 (9q34.13)* or *TSC2 (16p13.3)* genes, which encode hamartin and tuberin, respectively. Together, these proteins form a tumor suppressor complex that negatively regulates the mechanistic target of rapamycin (mTOR) signaling pathway. TSC is characterized by the development of benign hamartomas and cortical tubers [54]. An atypical but well-documented association with MMA has been recognized. In the cerebrovascular compartment, hyperactive mTOR signaling promotes the phenotypic switching and somatic hyperproliferation of VSMCs. However, the association with MMA is considered rare, and its actual prevalence remains unquantified as it has been, documented primarily through isolated case reports.

### 4.7. X-Linked Moyamoya

A distinct syndromic form of MMA, designated as Moyamoya disease 4 (MYMY4), is caused by mutations or deletions in the *BRCC3* gene located on chromosome Xq28 [55]. *BRCC3* encodes a deubiquitinating enzyme involved in DNA repair and inflammatory signaling. Patients with *BRCC3* aberrations typically present with a multisystemic phenotype including short stature, facial dysmorphisms, and hypogonadism, often referred to as “SHAM” syndrome (Severe Hemophilia A and Moyamoya) when deletions encompass the adjacent F8 gene [56,57].

### 4.8. Other Rare Syndromes

Grange syndrome is a rare autosomal recessive disorder characterized by severe, early-onset arterial steno-occlusive disease, bone dysplasia, syndactyly, and learning disabilities. It is caused by biallelic loss-of-function mutations in the *YY1AP1* gene (1q22), which encodes YY1-associated protein 1 [58]. *YY1AP1* plays a vital role in maintaining the transcriptional program of differentiated VSMCs. Its deficiency leads to a hyperproliferative VSMC state and robust intimal hyperplasia, frequently culminating in MMA. Steno-occlusive MMA is a hallmark and highly penetrant feature of this syndrome, reported in the majority (>80%) of patients described in the current literature [59].

Majewski Osteodysplastic Primordial Dwarfism Type II (MOPD II) is caused by mutations in the *PCNT* gene (21q22.3), which encodes pericentrin, a critical structural component of the centrosome [60]. It is an autosomal recessive disorder characterized by severe pre- and postnatal growth restriction, skeletal dysplasia, and marked microcephaly. Over 50% of patients are reported to develop severe neurovascular complications, including MMS or intracranial aneurysms, by early adulthood [61]. The underlying mechanism linking centrosomal dysfunction to MMA remains under active investigation, but it is hypothesized to involve impaired vascular endothelial cell migration and dysregulated mitotic spindle orientation during angiogenesis [61].

Schimke Immuno-Osseous Dysplasia (SIOD) is a highly penetrant autosomal recessive condition caused by mutations in the *SMARCAL1* gene (2q35), presenting with spondyloepiphyseal dysplasia, renal dysfunction (focal segmental glomerulosclerosis), and T-cell immunodeficiency [62]. *SMARCAL1* encodes a DNA annealing helicase involved in replication fork restart and DNA damage repair; defective DNA repair mechanisms in endothelial cells and VSMCs may trigger premature cellular senescence and aberrant vascular remodeling [63]. A subset of SIOD patients develops severe, early-onset MMA [63].

Alagille Syndrome (ALGS) is primarily caused by mutations in the Notch signaling pathway genes, *JAG1* or *NOTCH2*. While ALGS is characterized by multisystem involvement, the manifestation of classic MMA is rare, occurring in less than 1% of cases [64,65], and it is typically part of a broader, more heterogeneous spectrum of cerebral arteriopathies rather than an isolated finding.

Finally gain-of-function variants in *ANO1* (Anoctamin 1), which encodes a calcium-activated chloride channel, have been linked to a unique MMS phenotype [66] often featuring atypical progression into the posterior cerebral circulation.

## 5. Discussion

Moyamoya angiopathy is no longer considered a localized East Asian condition but is now recognized as a complex, multisystemic genetic spectrum. While RNF213 remains the major susceptibility gene in Asian populations, its low penetrance and the existence of RNF213-negative cases, particularly in Western cohorts, highlight the necessity of looking “beyond” this locus. Current research increasingly focuses on genes regulating the extracellular matrix, angiogenesis, and vascular inflammation, suggesting that the impact of these loss-of-function variants dictates a specific evolutionary trajectory of the disease.

Longitudinal data spanning 15 years indicate that the presence of the *RNF213* variant is a potent predictor of stenosis progression in the intracranial arteries, even in the absence of traditional cardiovascular risk factors [67]. This genetic predisposition appears to be a critical driver in the transition from isolated middle cerebral artery steno-occlusive disease to a full-blown MMD phenotype, suggesting that *RNF213* deficiency compromises the vessel’s ability to maintain structural integrity under hemodynamic stress [68].

Data from experimental models provide further insight into the functional consequences of *RNF213* loss of function, although these phenotypes often diverge from human clinical presentation. In murine models, *RNF213* deficiency tends to promote excessive microvascular branching and pathological angiogenesis rather than the hallmark stenosis of major intracranial arteries. Similarly, studies in zebrafish have revealed aberrant vessel sprouting and disorganized vascular networks, suggesting that *RNF213* loss primarily drives small-vessel remodeling [69,70,71,72].

At the cellular level, *RNF213* is predominantly expressed in endothelial cells; its knockdown enhances proliferation and migration through the activation of the Hippo pathway effectors YAP/TAZ and the upregulation of VEGFR2-dependent signaling [69,70,71,72]. These findings underscore a molecular drive toward aberrant vascular remodeling that remains a consistent hallmark of MMA bridging the gap between experimental observations and clinical reality [73]. Collectively, *RNF213* dysfunction appears to establish a “permissive” endothelial state that favors maladaptive angiogenic remodeling, from which progressive intimal expansion and cerebrovascular stenosis may subsequently emerge [71]. The *RNF213* p.R4810K variant is also associated with non-MMD intracranial arterial stenosis (ICAS) [74], pulmonary arterial hypertension [75] and systemic stenosis of the coronary, renal, and mesenteric arteries [76,77]. Thus, RNF213 stands as a central molecular determinant of MMA (Figure 1), linking an atypical ubiquitin ligase architecture to dysregulated endothelial remodeling.

The identification of diverse monogenic drivers in Western and *RNF213*-negative cohorts has redefined our comprehension of the disease. A central emerging theme is the role of VSMC dysfunction. Mutations in genes such as *ACTA2*, *MYH11*, and *DIAPH1* demonstrate how disruptions in the contractile apparatus and actin cytoskeleton drive pathological remodeling. This differs from the “proliferative” mechanism observed in RASopathies, where hyperactivation of the RAS/MAPK pathway triggers intimal hyperplasia. Additionally, the association between interferonopathies and Moyamoya highlights the impact of chronic neuro-inflammation on vascular homeostasis.

These advancements have prompted a paradigm shift, transitioning the classification of MMA from a purely vascular occlusive disorder to a neurocristopathy. This hypothesis suggests the primary defect lies in the embryological origin of the cranial vasculature, specifically involving the neural crest cells (NCCs) [18,78,79]. The anatomical predilection of MMA for the terminal portion of the ICA can be explained by its unique chimeric assembly: the proximal segment originates from the third aortic arch (mesoderm-derived) while the distal segment is derived from the NCCs, representing a locus minoris resistentiae where developmental errors in NCC migration manifest as progressive occlusion. This “neurocristopathy model” provides a unifying framework for the high frequency of associated craniofacial, cardiac, and endocrine anomalies seen in MMS.

Moreover, the convergence of ACTA2, MYH11, and DIAPH1 onto the same functional endpoint—VSMC dysfunction—suggests a unifying developmental origin. Given that the VSMCs of the terminal ICA are derived from the NCCs, these genetic defects likely induce a fundamental structural fragility in the NCC-derived ectomesenchyme. In this integrated model, the TGF-β-induced “proliferative stenosis” and the “dissection phenotype” represent two manifestations of the same underlying failure of NCC-derived lineages to maintain vascular homeostasis under hemodynamic stress.

Current international guidelines exhibit variations in genetic testing indications. Table 3 provides a comparative summary of current international recommendations for genetic screening. The Japanese Research Committee guidelines emphasize diagnostic utility in familial cases [80], while the AHA/ASA [81] and European Stroke Organization (ESO) guidelines endorsed by the Vascular European Reference Network (VASCERN) [82] prioritize genetic evaluation when “red flags” are present, such as early onset (before age 5), syndromic features, or atypical radiologic patterns. Furthermore, these guidelines underscore that any genetic testing should be strictly performed alongside formal genetic counseling of Moyamoya care.

Despite these advancements, several limitations persist. First, the literature is heavily skewed toward East Asian populations, potentially underestimating genetic drivers in Western or African cohorts. Second, many rare variants identified in MMS cases lack rigorous functional validation in patient-derived cellular models or organoids. Future research must prioritize large-scale, international multi-omic collaborations to integrate genomic, transcriptomic, and proteomic data. For non-Asian patients or those with syndromic features, a Moyamoya-specific Next-Generation Sequencing panel is recommended, as this “genetics-first” approach is fundamental for identifying specific genotypes in seemingly sporadic cohorts [83,84]. In cases where panels are inconclusive, Whole-Exome or Whole-Genome Sequencing is indicated [85].

In conclusion, Genotype-informed management represents the future of MMA care. For instance, patients with ACTA2 mutations require frequent systemic imaging for aortic risks, while understanding the inflammatory drivers in SAMHD1 patients could enable targeted therapies with JAK inhibitors. Moving beyond traditional revascularization, the expansion of our genetic map aims to provide every patient with a personalized strategy addressing the underlying biological cause of their disease.

## Figures and Tables

**Table 1:** Summary of non-RNF213 genes associated with Moyamoya Syndrome.

Gene	Biological Pathway	Variant Type	Pathogenic Mechanism	Genotype–Phenotype Correlation
*ACTA2*	VSMC Contraction and Cytoskeleton	Missense (typically p.R179 variants	Defective α-smooth muscle actin assembly	MMS; multisystemic smooth muscle dysfunction
*MYH11*	VSMC Contraction and Cytoskeleton	Missense variant autosomal dominant	Disrupted myosin assembly and contractility	MMS; familial thoracic aortic aneurysms and dissections, patent ductus arteriosus.
*DIAPH1*	RhoA GTPase signaling pathway	Missense mutation (Arg1213)	Loss of actin polymerization and microtubule stability	MMS; Seizures, Cortical Blindness, and Microcephaly Syndrome
*GUCY1A3*	NO-cGMP Signaling	Loss-of-function	Impaired cGMP synthesis and VSMC hyperplasia	Early onset MMS and achalasia
*SAMHD1*	IFN pathway	Loss-of-function	IFN-mediated inflammation immune regulation	MMS; Early onset stroke, multifocal stenosis of major intracranial arteries
*MTHFR*	Homocysteine metabolism	SNP rs9651118		MMS; Hyperomocysteinemia

Legend: MMS: Moyamoya Syndrome; VSMC: Vascular Smooth Muscle Cell; NO: Nitric Oxide.

**Table 2:** Syndromic associations of Moyamoya Syndrome.

Condition	Gene/Genes	Pathway	Main Vascular Mechanism
Down syndrome	*DYRK1A*, *IFNAR1/2*, *IFNGR2*, *IL10RB*	Interferon signaling	VSMC dysfunction, pro-fibrotic remodeling
RASopathies	*PTPN11*, *SOS1*, *BRAF*	RAS/MAPK	VSMC proliferation, endothelial dysregulation
Sickle cell disease	*HBB*	Hemolysis, inflammation	Endothelial activation, collateral formation
Marfan syndrome	*FBN1*	TGF-β signaling	ECM remodeling, intimal fibrosis
Pseudoxanthoma elasticum	*ABCC6*	PPi metabolism	Elastic fiber calcification, intimal proliferation
Neurofibromatosis type 1	*NF1*	RAS/MAPK	VSMC hyperproliferation, intimal hyperplasia
Tuberous sclerosis	*TSC1*, *TSC2*	mTOR signaling	VSMC proliferation, vessel wall thickening
X-linked Moyamoya	*BRCC3*	DNA repair, inflammation	Multisystem vascular involvement
Grange syndrome	*YY1AP1*	Transcriptional regulation	Severe VSMC proliferation, stenosis
Alagille syndrome	*JAG1*, *NOTCH2*	Notch signaling	Vascular fragility, arterial stenosis

Legend: VSMC: Vascular Smooth Muscle Cell; ECM: Extracellular Matrix; PPi: Inorganic Pyrophosphate; TGF-β: Transforming Growth Factor-β.

**Table 3:** International clinical guidelines for genetic testing in MMA.

Guideline/Organization	Core Recommendations	Target Population	“Red Flags” for Genetic Evaluation
Japanese Research Committee	Emphasizes the diagnostic utility of genetic screening to confirm susceptibility and support clinical diagnosis.	Familial cases and suspected idiopathic MMD.	Positive family history of MMD; East Asian ancestry.
American Heart Association	Recommends prioritizing genetic evaluation when syndromic features or specific clinical markers are present.	Pediatric cases and non-Asian cohorts.	Early onset (before age 5); facial dysmorphisms; intellectual disability; associated organ malformations.
European Stroke Organization	Recommends a targeted approach; emphasizes that genetic testing must be performed alongside formal genetic counseling.	Pediatric cases; patients with a positive family history; suspected syndromic forms.	Involvement of the posterior circulation; presence of extracranial stenoses (e.g., renal or mesenteric arteries).

## Data Availability

No new data were created or analyzed in this study. Data sharing is not applicable to this article.
